# Microbial strategies for drought stress mitigation- a sustainable frontier in plant resilience

**DOI:** 10.3389/fpls.2025.1740879

**Published:** 2026-01-06

**Authors:** Kumar D. Gahlot

**Affiliations:** Department of Molecular Biology and Umeå Centre for Microbial Research (UCMR), Umeå University, Umeå, Sweden

**Keywords:** microbial strain development, microbial diversity, multi-omics analysis, signaling pathways, sustainable agricultural applications

## Abstract

Drought stress is a major constraint on global agriculture, exacerbated by climate change and increasing water scarcity. Conventional strategies such as breeding and genetic engineering have improved drought tolerance in crops, yet their scalability and adaptability remain limited. Microbial interventions, particularly those involving beneficial plant-associated microorganisms, offer a sustainable and complementary approach to enhance plant resilience under water-deficit conditions. This opinion article explores microbial strategies for drought mitigation, emphasizing the role of *Rhizobium* strains, digested distillery spent wash, and multi-omics technologies. Recent studies demonstrate that developed *Rhizobium* strains significantly improve soil fertility, nodulation, and nitrogen fixation in legumes, contributing to higher yields and better soil health in drought-prone regions. Similarly, the application of digested distillery spent wash in chickpea (*Cicer arietinum*) enhances nutrient uptake, photosynthetic activity, and drought tolerance. Advances in genomics, transcriptomics, proteomics, and metabolomics have revealed complex plant–microbe interactions, identifying microbial metabolites and signaling pathways that activate drought-responsive genes and osmo-protective mechanisms. Despite these promising findings, challenges persist in translating laboratory results to field conditions due to soil heterogeneity and microbial competition. Precision microbiome engineering, informed by multi-omics data, and the development of tailored microbial consortia represent a transformative frontier for sustainable agriculture. By integrating ecological complexity with technological innovation, microbial strategies can reduce chemical inputs, promote regenerative practices, and build resilient agroecosystems. This article advocates elevating microbes from supporting roles to central players in addressing drought stress and ensuring global food security.

## Perspective

As climate change worsens drought stress across agricultural landscapes, the urgency to develop resilient cropping systems has never been greater. While traditional breeding and genetic engineering have helped create drought-tolerant crops, microbial strategies- especially those involving beneficial plant-associated microorganisms- offer a complementary and sustainable solution (Vurukonda et al., 2016; Naylor et al., 2017; Allito et al., 2021; Shah et al., 2024; Sharma et al., 2025). In my opinion, integrating microbial interventions, particularly those guided by multi-omics approaches (Kumar et al., 2023; Jain et al., 2024), represents a transformative frontier in drought mitigation.

Recent studies have shown the effectiveness of developed *Rhizobium* strains in enhancing soil fertility and legume yields, especially in regions like Haryana, India (Shah et al., 2024). These strains not only boost nodulation and nitrogen fixation but also improve overall soil health, which is vital during drought conditions when nutrients are less available. Additionally, the use of digested distillery spent wash has demonstrated encouraging effects on chickpea (*Cicer arietinum*), enhancing nodulation, nutrient absorption, and photosynthesis- key traits that support drought tolerance (Gahlot et al., 2011).

What excites me most is the increasing adoption of multi-omics technologies- such as genomics, transcriptomics, proteomics, and metabolomics- to explore the complex molecular dialogue between plants and microbes. These tools offer a comprehensive view of how microbial communities impact plant stress responses, nutrient cycling, and immune regulation (Kumar et al., 2023; Jain et al., 2024). For instance, transcriptomic analyses have shown how microbial inoculants can activate drought-responsive genes, while metabolomics has identified microbial metabolites that function as osmo-protectants or signaling molecules.

Despite these advances, converting lab-based findings into practical field applications remains challenging. Soil heterogeneity, microbial competition, and environmental variability often reduce the effectiveness of inoculants. However, tailored microbial consortia- designed using omics-informed selection- could surmount these barriers by providing synergistic benefits across various agroecological zones.

The future of microbial drought mitigation depends on embracing ecological complexity and integrating technology. We need to move beyond single-strain inoculants and focus on precision microbiome engineering, backed by long-term field trials and farmer-participatory research. Microbial strategies are not a cure-all, but they align with regenerative agriculture principles, decrease reliance on chemicals, and build resilience in both plants and ecosystems.

**Figure 1:**
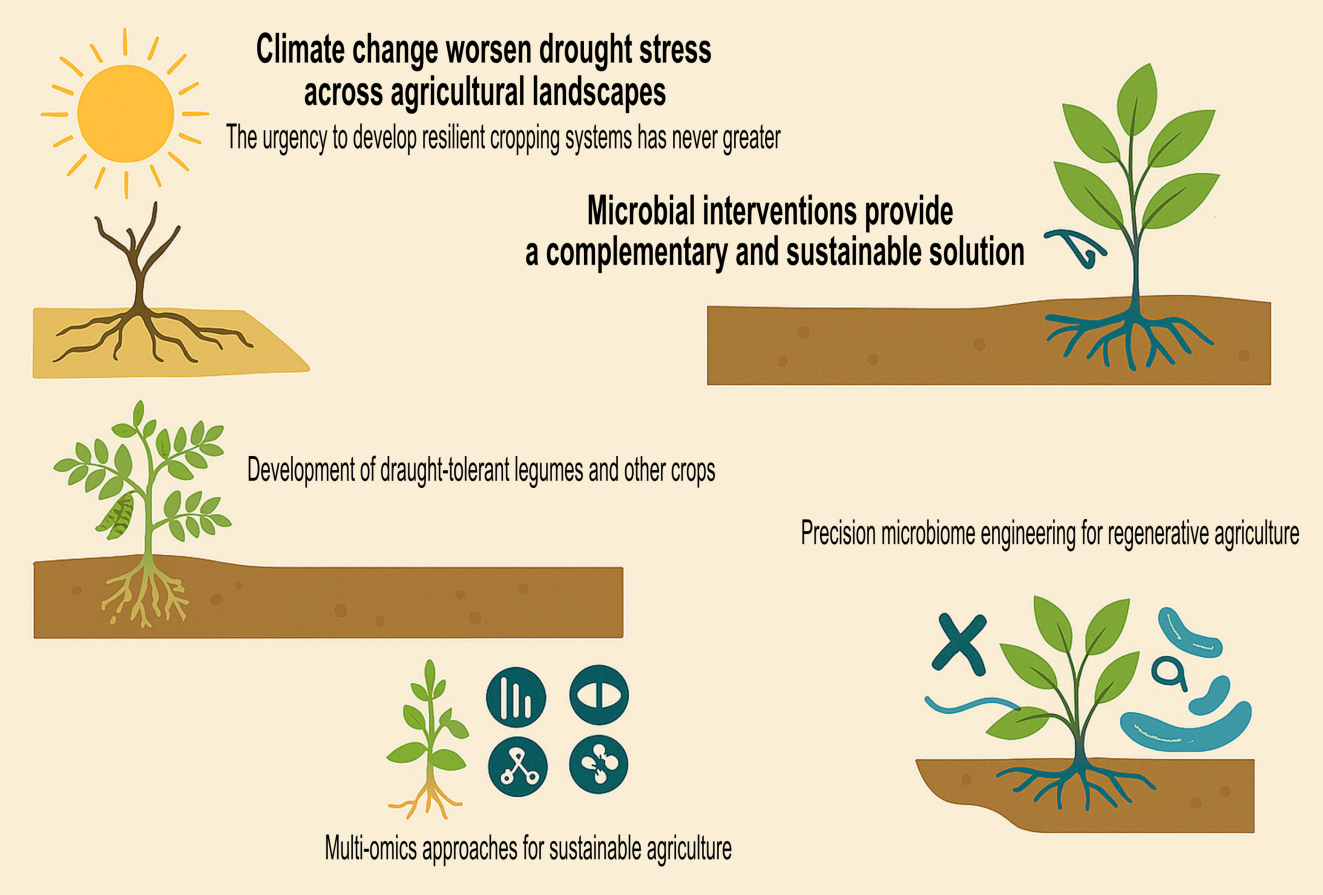
Conceptual overview of microbial strategies for drought resilience. The illustration emphasizes key themes: (i) the urgency of addressing climate change-induced drought stress in agriculture; (ii) microbial interventions as sustainable solutions; (iii) drought-tolerant legumes supported by *Rhizobium* strains; (iv) the role of digested distillery spent wash in enhancing chickpea nodulation and nutrient uptake; (v) integrating multi-omics approaches (genomics, transcriptomics, proteomics, metabolomics) to understand plant–microbe interactions; and (vi) precision microbiome engineering for improved crop resilience under water-deficit conditions.

As we face the realities of climate change, it is time to elevate microbes from supporting roles to leading players in the story of drought resilience. My conceptual overview of microbial strategies for drought resilience in various cropping systems for sustainable agriculture is shown in **Figure 1**.

## Data Availability

Publicly available datasets were analyzed in this study. This data can be found here: Cited References are Open-Access.
